# Lanthanide single-molecule magnets with high anisotropy barrier: where to from here?

**DOI:** 10.1093/nsr/nwac194

**Published:** 2022-09-20

**Authors:** Zhenhua Zhu, Jinkui Tang

**Affiliations:** State Key Laboratory of Rare Earth Resource Utilization, Changchun Institute of Applied Chemistry, Chinese Academy of Sciences, China; University of Chinese Academy of Sciences, China; State Key Laboratory of Rare Earth Resource Utilization, Changchun Institute of Applied Chemistry, Chinese Academy of Sciences, China; University of Science and Technology of China, China

## Abstract

High-temperature lanthanide single-molecule magnets with large coercive fields are being pursued.

Lanthanide complexes with magnetic hysteresis known as lanthanide single-molecule magnets (Ln-SMMs) are promising candidates for high-density information storage and quantum technologies. Due to the predominantly electrostatic nature of 4f–ligand interactions, the coordination geometry of lanthanide complexes is contingent upon the steric constraints. The oblate–prolate model suggests that axial coordination environment with linear arrangement of more repulsive ligands is suitable for oblate ions, e.g. Dy^3+^, while equatorially coordinating geometry is preferable for prolate ions, e.g. Er^3+^ [1]. In terms of synthesis, by contrast to the placement of more repulsive ligands along a single axis, it is more challenging to achieve the arrangement along a plane. Therefore, few ligands have been discovered to produce effective equatorial crystal field (CF) and the most popular one in constructing Er-based SMMs is dianion of substituted cyclooctatetraenyl (Fig. 1A and C). In contrast, various N-/O-donor ligands rooted in Werner-type coordination chemistry, e.g. Schiff-base and phenolate/alkoxide, are employed to enhance the magnetic anisotropy of Dy^3+^ complexes (Fig. 1A). In addition, for powerful Tb-based SMMs, high molecular symmetry is necessary to guarantee ±*M_J_* degeneracy, which is indeed difficult in synthesis due to the large radii and variable coordination patterns of Ln^3+^ ions. Fortunately, the Kramers nature of Dy^3+^ ensures a bistable ground state regardless of the coordination geometry. Furthermore, the coordination geometry does not need to be perfectly axial to stabilize the magnetic ground state of *M_J_* = ±15/2 of Dy^3+^ ions, i.e. allowing the existence of weak transverse CF. Therefore, some low-symmetry Dy-based SMMs with high anisotropy barrier (*U*_eff_, the energy required to convert an SMM back into a simple paramagnet) were constructed by the *predominant bond* that usually refers to the shortest Dy–X chemical bond (X is the closest bonding atom) (Fig. 1A and B). To sum up, the design of Ln-SMMs with high *U*_eff_ is almost exclusively driven by static CF considerations.

**Figure 1.**
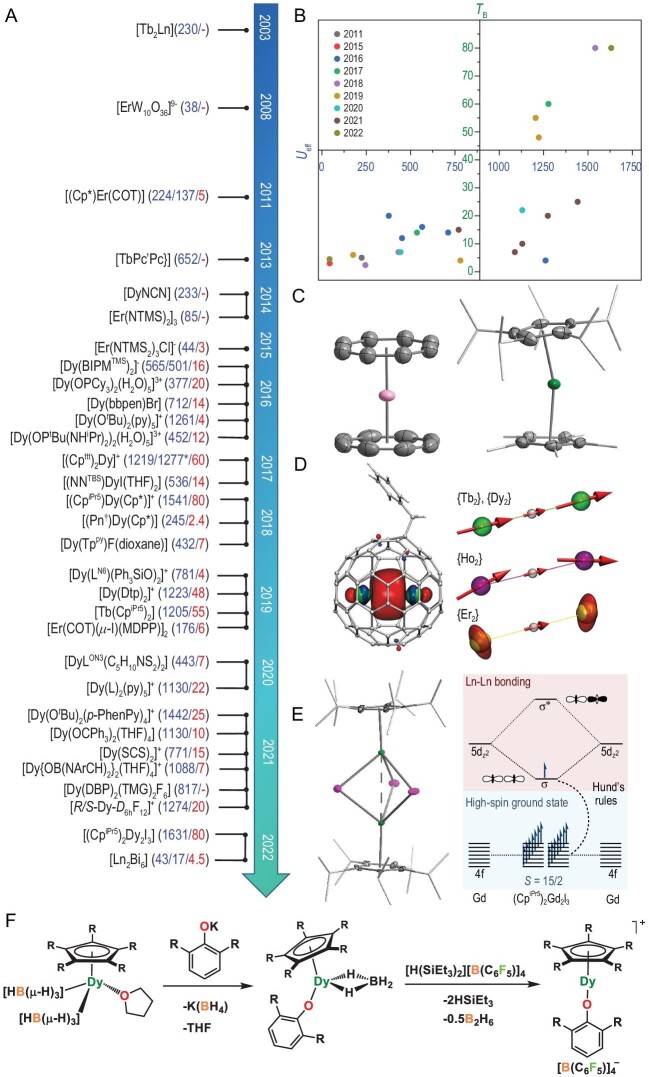
(A) Timeline of the development of high-performance Ln-SMMs [numbers in blue and red are *U*_eff_ (cm^−1^) and *T*_B_ (K), respectively. Asterisk indicates the two independent studies of the same compound.]. (B) Relationship between *U*_eff_ and *T*_B_ of the compounds in Fig. 1A. (C) Solid structures of [Er(COT)_2_]^−^ (left) [2] and [(Cp^iPr5^)Dy(Cp*)]^+^ (right) [3]. (D) Singly occupied Ln–Ln molecular orbital (left) and orientation of Ln magnetic moments in Ln_2_@C_80_(CH_2_Ph) (right) [4]. (E) Solid structure of (Cp^iPr5^)_2_Ln_2_I_3_ (left) and molecular orbital diagram for (Cp^iPr5^)_2_Gd_2_I_3_ (right). Adapted from [5] with permission from American Association for the Advancement of Science. (F) A possible synthesis route for half-sandwich dysprosium metallocene.

However, *U*_eff_ can only provide information on magnetic relaxation in the high-temperature regime corresponding to the Orbach mechanism, while the blocking temperature (*T*_B_, the temperature at which the relaxation time of the magnetization equals the characteristic time of the experiment) is a more informative parameter involving all sources of demagnetization. As we all know, the Raman and quantum tunneling processes together prevent *T*_B_ landing in the exponential relaxation regime. Over the last several years, air-sensitive dysprosium metallocene cations, [Dy(Cp^R^)_2_]^+^, have emerged as clear front-runners in the search for high-temperature Ln-SMMs. They possessed both large *U*_eff_ and high *T*_B_ benefitting from the near-perfect axiality and the constrained metal–ligand vibrational modes, among which compound [(Cp^iPr5^)Dy(Cp*)][B(C_6_F_5_)_4_] (Cp^iPr5^ = penta-iso-propylcyclopentadienyl, Cp* = pentamethylcyclopentadienyl), exhibits *T*_B_ up to 80 K (Fig. 1C), exceeding the boiling point of liquid nitrogen [3]. In addition, it is noteworthy that Dy^3+^ corannulene complexes are expected to have both improved air stability and excellent SMM properties [6].

The most notable exceptions beyond the above design criteria are radical-bridged Ln-SMMs, which usually display a modest *U*_eff_ but a high *T*_B_ and/or a huge coercive field due to the strong exchange coupling. The ultimate realization of such exchange coupling might be completed by a single-electron Ln–Ln bond. The confined space in fullerene provides a unique opportunity for building such a metal–metal bond. For example, Tb_2_@C_80_(CH_2_Ph) showed *U*_eff_ and *T*_B_ of 555 cm^−1^ and 28 K, benefitting the combination of strong single-ion magnetic anisotropy and exchange coupling from a single-electron Tb–Tb bond (Fig. 1D) [4]. Furthermore, *ab initio* calculations revealed a higher *U*_eff_ and a stronger exchange coupling in Tb_2_@C_59_N [7]. However, the coulombic repulsion between the lanthanide spins in fullerene is stronger than the bonding interaction, so it is necessary to find a more conventional way to construct a single-electron Ln–Ln bond, which was recently completed in mixed-valence dilanthanide complexes, (Cp^iPr5^)_2_Ln_2_I_3_ (Fig. 1E) [5]. The singly occupied Ln–Ln σ-bonding orbital of 5d_z_^2^ parentage enables the well-isolated high-spin ground state even at room temperature. The 4f-σ coupling constant in (Cp^iPr5^)_2_Gd_2_I_3_ reaches 387(4) cm^−1^. Compound (Cp^iPr5^)_2_Dy_2_I_3_ exhibited *U*_eff_ and 100 s-*T*_B_ of 1631(25) cm^−1^ and 72 K, respectively, as well as an enormous coercive magnetic field with a lower bound of 14 T at 60 K. Note that all of them are the largest values for any reported Ln-SMMs.

The above advances demonstrate that the most important figure of merit for SMMs has evolved from *U*_eff_ to *T*_B_. In particular, the dominance of the Cp ligand in enhancing *T*_B_ has garnered considerable attention, although *ab initio* spin dynamics methodology revealed that [(Cp^iPr5^)Dy(Cp*)][B(C_6_F_5_)_4_] has reached the upper limit to the *U*_eff_ of [Dy(Cp^R^)_2_]^+^, which means the predominant limiting mechanism for a further increase in *T*_B_ is the Orbach process [8]. Given the strong interaction between Dy^3+^ and phenolate, we proposed a half-sandwich dysprosium metallocene (Fig. 1F), which is expected to break this limit by virtue of the combination of large CF splitting from phenolate and unique vibrational modes of the Cp ligand. Besides, the molecular vibrations also need to be taken into account as they play a prominent role in under-barrier magnetic relaxations by coupling to spin states or an electrostatic polarization effect [9]. In general, detrimental vibrational modes can be blocked by decoupling spin from vibration in the chemical approach or choosing a substrate with a low phonon density of state, e.g. MgO [10]. It is noteworthy that in addition to the requirements in physical properties of SMMs, their stability under ambient conditions is also crucial to realize the proposed applications, so a complementary stream of research has focused on improving SMM performance in air-stable lanthanide macrocycles.

## References

[1] Rinehart JD , Long JR. Chem Sci 2011; 2: 2078–85. 10.1039/c1sc00513h

[2] Ungur L , Le Roy JJ, Korobkov I et al. Angew Chem Int Ed 2014; 53: 4413–7. 10.1002/anie.201310451 24652777

[3] Guo F-S , Day BM, Chen Y-C et al. Science 2018; 362: 1400–3. 10.1126/science.aav0652 30337456

[4] Liu F , Velkos G, Krylov DS et al. Nat Commun 2019; 10: 571. 10.1038/s41467-019-08513-6 30718550 PMC6362165

[5] Gould CA , Mcclain KR, Reta D et al. Science 2022; 375: 198–202. 10.1126/science.abl5470 35025637

[6] Sharma T , Singh MK, Gupta R et al. Chem Sci 2021; 12: 11506–14. 10.1039/D1SC03160K 34667554 PMC8447237

[7] Dey S , Rajaraman G. Chem Sci 2021; 12: 14207–16. 34760206 10.1039/d1sc03925c PMC8565386

[8] Reta D , Kragskow JGC, Chilton NF. J Am Chem Soc 2021; 143: 5943–50. 10.1021/jacs.1c01410 33822599

[9] Briganti M , Santanni F, Tesi L et al. J Am Chem Soc 2021; 143: 13633–45. 10.1021/jacs.1c05068 34465096 PMC8414553

[10] Donati F , Rusponi S, Stepanow S et al. Science 2016; 352: 318–21. 10.1126/science.aad9898 27081065

